# COVID-19 and Congenital Heart Disease: Results from a Nationwide Survey

**DOI:** 10.3390/jcm9061774

**Published:** 2020-06-08

**Authors:** Jolanda Sabatino, Paolo Ferrero, Massimo Chessa, Francesco Bianco, Paolo Ciliberti, Aurelio Secinaro, Lilia Oreto, Martina Avesani, Valentina Bucciarelli, Giuseppe Calcaterra, Maria Pia Calabrò, Maria Giovanna Russo, Pier Paolo Bassareo, Paolo Guccione, Ciro Indolfi, Giovanni Di Salvo

**Affiliations:** 1 Department of Medical and Surgical Sciences, “Magna Graecia” University, 88100 Catanzaro, Italy; jolesbt@hotmail.it (J.S.); indolfi@unicz.it (C.I.); 2 Pediatric cardiology and ACHD, Cardiovascular Department, ASST Papa Giovanni 23, 24127 Bergamo, Italy; pferrero@asst-pg23.it; 3 ACHD UNIT-Pediatric and Adult Congenital Heart Centre, IRCCS-Policlinico San Donato, 20097 Milan, Italy; massichessa@yahoo.it; 4 Pediatric and Congenital Cardiology Unit, Azienda Ospedaliero Universitaria Ancona, 60126 Ancona, Italy; dr.francescobianco@gmail.com (F.B.); valentina_bucciarelli@yahoo.it (V.B.); 5 Pediatric Cardiology and Cardiac Surgery Department, Bambino Gesù Children’s Hospital, IRCCS, 00146 Rome, Italy; paolo.ciliberti@opbgmail.onmicrosoft.com (P.C.); paolo.guccione@opbg.net (P.G.); 6 Department of Imaging, Advanced Cardiovascular Imaging Unit, Bambino Gesù Children’s Hospital, IRCCS, 00146 Rome, Italy; aurelio.secinaro@opbg.net; 7 Mediterranean Pediatric Cardiology Center, Bambino Gesù Pediatric Hospital, 98039 Taormina (ME), Italy; liliaoreto@hotmail.com; 8 Department of Women’s and Children’s Health, University of Padua, 35122 Padua, Italy; martiaavesani1@gmail.com; 9 Department of Cardiology, University of Palermo, 90133 Palermo, Italy; peppinocal7@gmail.com; 10 Department of Human Pathology of Adulthood and Childhood, University of Messina, 98125 Messina, Italy; mpcalabro@unime.it; 11 Department of Cardiology, Luigi Vanvitelli University of Naples, Monaldi Hospital, 80131 Naples, Italy; mariagiovanna.russo@unina2.it; 12 University College of Dublin, Mater Misericordiae University Hospital, Dublin 4, Ireland; piercard@inwind.it

**Keywords:** congenital heart disease, COVID-19, cardiovascular complications

## Abstract

Background. The pandemic of Novel Coronavirus Disease 2019 (COVID-19) is challenging, given the large number of hospitalized patients. Cardiovascular co-morbidities are linked to a higher mortality risk. Thus, patients with Congenital Heart Disease (CHD) might represent a high-risk population. Nevertheless, no data about them are available, yet. Hence, we conducted a nationwide survey to assess clinical characteristics and outcomes in patients with congenital heart disease affected by COVID-19. Methods and Results. This is a multi-centre, observational, nationwide survey, involving high-volume Italian CHD centres. COVID-19 diagnosis was defined as either “clinically suspected” or “confirmed”, where a severe acute respiratory syndrome coronavirus 2 (SARS-CoV2) test had been performed and was positive. Cardiovascular comorbidities were observed among adult patients—atrial fibrillation (seven; 9%), hypertension (five; 7%), obesity (seven; 9%) and diabetes (one; 1%)—but were absent among children. Cardiovascular complications were mainly observed in the “confirmed” COVID-19^+^ group, consisting of heart failure (9%), palpitations/arrhythmias (3%), stroke/TIA (3%) and pulmonary hypertension (3%). Cardiovascular symptoms such as chest pain (1%), myocardial injury (1%) and pericardial effusion (1%) were also recorded. On the contrary, CHD patients from the clinically suspected COVID-19 group presented no severe symptoms or complications. Conclusions. Despite previous reports pointing to a higher case-fatality rate among patients with cardiovascular co-morbidities, we observed a mild COVID-19 clinical course in our cohort of CHD patients. Although these results should be confirmed in larger cohorts to investigate the underlying mechanisms, the findings of low cardiovascular complications rates and no deaths are reassuring for CHD patients.

## 1. Background

Facing *Novel Coronavirus Disease 2019* (COVID-19) is complex, given the high transmission rate (R_0_ = 1.4–3.8) and the large number of hospitalized patients, often needing intensive care and ventilation support [1,2,3].

According to initial reports from Wuhan and the Hubei region, patients with cardiovascular co-morbidities are at higher risk of morbidity and mortality [4,5,6]. A meta-analysis of observational studies [6] showed the incidences of hypertension, cerebrovascular diseases and diabetes were higher in Intensive Care Unit (ICU)/severe cases (28.8%, 16.7% and 11.7%, respectively) than in their non-ICU/severe counterparts (14.1%, 6.2% and 4.0%, respectively) [6]. Additionally, a single-centre study by Guo et al. [5], on hospitalized COVID-19 patients, stated that the case fatality rate was higher (13.3%) for patients with underlying cardiovascular comorbidities compared to those without (7.6%), with a dramatic increase (69.4%) in patients with underlying cardiovascular comorbidities experiencing myocardial injury also during hospitalization.

For this reason, subjects that might be considered at particularly high risk should comprise patients with Congenital Heart Disease (CHD) [7,8], both children and adults.

CHD is the most common and global inborn defect. With an increasing life expectancy amongst them, CHD patients are susceptible to acquired cardiovascular and other diseases and to environmental threats, including infectious diseases [9].

This risk might be further multiplied if CHD is associated with additional comorbidities—lung disease, pulmonary hypertension and heart failure—or in presence of complex congenital heart disease [7,8,9,10,11].

Nevertheless, several knowledge gaps remain around this association, and to date, there are no available published studies on COVID-19 patients, both children and adults, with congenital heart disease.

For these reasons, we collected data from a multi-centre, observational, nationwide survey in order to evaluate clinical characteristics and the prevalence of adverse outcomes in patients with congenital heart disease affected by COVID-19.

## 2. Methods

We conducted a multi-centre, cross-sectional, observational, nationwide survey aimed at evaluating consecutive patients with congenital heart disease admitted to Italian Congenital Heart Disease Units affiliated and associated with the CHD working group of the Italian Society of Cardiology, during a six-week period of the initial COVID-19 outbreak in Italy: 21 February–4 April. All patients admitted with CHD, who were diagnosed with COVID-19 and either treated and discharged or who died during hospitalization in the 6-week window, were included independently of their age. COVID-19 was diagnosed according to the guidelines of the World Health Organization [12]. Nasopharyngeal swabs were acquired during hospitalization. Real-time polymerase chain reaction tests were applied to diagnose COVID-19, according to recommended protocols. Infection was established as whether at least two positive test results were observed [13].

Data were reported for two groups of patients: (i) confirmed diagnosis of COVID-19 (CHD-Covid-19+), if they presented positive nasopharyngeal swabs with suggestive clinical symptoms; and (ii) suspected diagnosis of COVID-19 (suspCHD-Covid-19), if they were exposed to contagion and presented a suggestive history and clinical symptoms, in the absence of confirmation from nasopharyngeal swabs, where they were not performed or not available.

### 2.1. Data Collection and Data Quality

The Congenital Heart Disease-Working Group of the Italian Society of Cardiology (Società Italiana di Cardiologia, SIC) invited all affiliated and associated Italian hospitals to get involved in the survey, including academic and non-academic hospitals with Congenital Heart Disease Units receiving CHD patients (Bergamo Hospital; Ancona Hospital; Policlinico San Donato of Milan; University of Padua; Bambino Gesù Children’s Hospital of Rome; Bambino Gesù Pediatric Hospital of Taormina; University of Messina; Monaldi Hospital of Naples, Italy).

Clinical data on patients admitted for the above-reported diagnosis were recorded, including epidemiological, demographical and clinical symptoms and complications, management and outcomes.

At each site, a coordinating investigator was in charge of screening consecutive patients admitted to the hospital, data collection and quality. Data were collected using report sheets at single centres. After collection, the participating centers submitted filled-in report sheets to the coordinating unit at Padua University that was in charge of transferring all the data into Excel-based electronic worksheets. The data were finally checked for missing or contradictory entries at the coordinating centre.

### 2.2. Cardiovascular Complications

Cardiovascular complications, as adjudicated through medical records, were: (a) palpitations/arrhythmias (defined as rapid ventricular tachycardia lasting more than 30 s), (b) chest pain, (c) myocardial injury (if serum levels of troponin T (TnT) were above the 99th percentile upper reference limit), (d) heart failure (HF) (defined by a physician diagnosis of HF, which included documentation of one of the following: impaired LV systolic or diastolic dysfunction, pulmonary edema/congestion, dilated ventricles or reduced right ventricular function), (e) stroke/TIA (defined according to the AHA Definition [14]), (f) pulmonary hypertension (increased >20 mmHg compared to baseline values), (g) pericardial effusion, and (h) respiratory failure (defined according to the Berlin Definition [15]).

Categorical variables are presented as frequency rates and percentages. Continuous variables are presented as mean and standard deviation (SD).

## 3. Results

**Figure 1:**
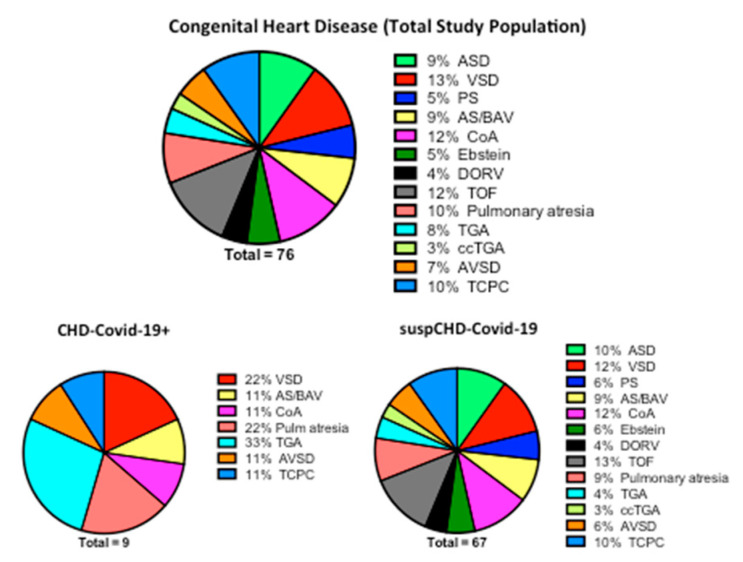
Congenital Heart Disease distribution on admission to high-volume Italian CHD centres with “clinically suspected” or “confirmed” COVID-19 diagnoses. CHD = Congenital Heart Disease; ASD = atrial septal defect; VSD = ventricular septal defect; PA = pulmonary stenosis; AS/BAV = aortic stenosis/bicuspid aortic valve; COA = coarctation of the aorta; DORV = double outlet right ventricle; TOF = tetralogy of Fallot; TGA = transposition of the great arteries; ccTGA = congenitally corrected transposition of the great arteries; AVSD = atrioventricular septal defect; TCPC = total cavopulmonary connection; CHD-Covid-19+ = confirmed diagnosis of COVID-19; suspCHD-Covid-19 = clinically suspected COVID-19 (no confirmation test available during the outbreak).

A total of eight high-volume CHD centres (Bergamo Hospital; Ancona Hospital; Policlinico San Donato of Milan; University of Padua; Bambino Gesù Children’s Hospital of Rome; Bambino Gesù Pediatric Hospital of Taormina; University of Messina; Monaldi Hospital of Naples, Italy) participated in data collection. Overall, 76 COVID-19-infected CHD cases were registered from 21 February to 4 April 2020 and were reported in this study, including four children (less 18 years old) and 72 adults. The distributions of the CHDs reported are listed in Figure 1.

The children group comprised two girls and two boys, with a mean age of 0.9 years (range: 2 months to 2 years). The adult group included 38 men and 34 women, with a mean age of 36.6 years (range: 21 to 76 years).

Looking at single subgroups, nine patients presented a confirmed diagnosis of COVID-19 and were included in the CHD-Covid-19+, while the remaining 67 had no microbiological confirmation (no test available during the outbreak) and were classified according to the WHO guidelines [12] (suspCHD-Covid-19). Underlying cardiovascular comorbidities and risk factors are summarised in Table 1. They were observed in adult patients—especially atrial fibrillation (seven; 9%), hypertension (five; 7%), obesity (seven; 9%) and diabetes (one; 1%)—but were virtually absent in the paediatric population.

### 3.1. Cardiovascular Complications

**Figure 2:**
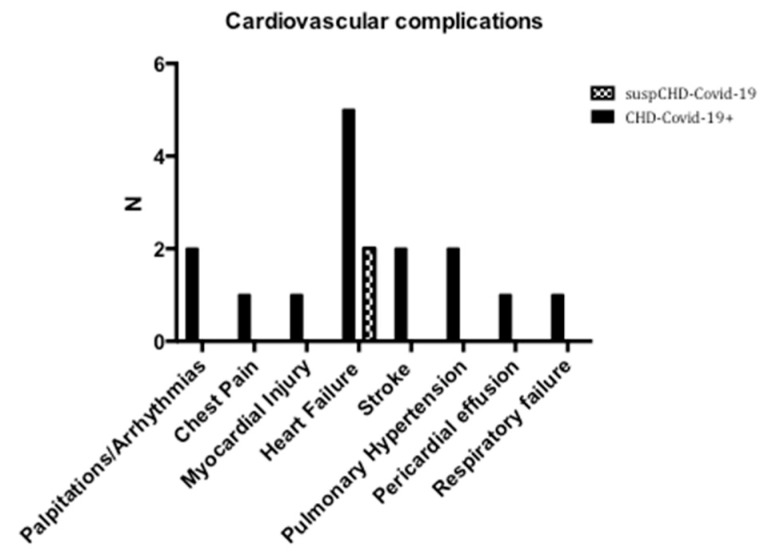
Cardiovascular complications of patients with congenital heart disease and CHD-Covid-19+ (confirmed diagnosis of COVID-19) or suspCHD-Covid-19 (clinically suspected COVID-19). N = number of cases.

Cardiovascular complications were mainly observed in the CHD-Covid-19+ group (Figure 2). The most represented cardiovascular complication, among the whole population collected for the survey, was heart failure (9%), followed by palpitations/arrhythmias (3%), stroke/TIA (3%) and pulmonary hypertension (3%). Chest pain (1%), myocardial injury (1%) and pericardial effusion (1%) were also recorded (Figure 2).

When CHD-Covid-19+ patients were considered alone (Table 2), heart failure was observed in 55% of the total cases, while palpitations/arrhythmias, stroke/TIA and pulmonary hypertension were observed in 22% of the cases. In one out of nine CHD-Covid-19+ patients were observed chest pain, myocardial injury or pericardial effusion.

Finally, one patient from the CHD-Covid-19+ group presented severe respiratory failure and was treated with C-PAP in ICU.

### 3.2. Clinical Management and Treatment

**Figure 3:**
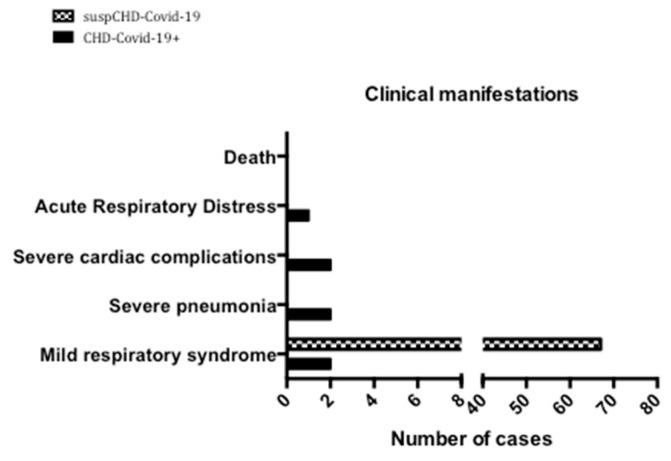
Clinical manifestations and outcome of patients with Congenital Heart Disease and “clinically suspected” or “confirmed” COVID-19 diagnosis.

All the patients from the suspCHD-Covid-19 group presented with mild infection, without any severe symptoms or complications (Figure 3). They recovered within 1 to 2 weeks with prompt symptomatic treatment. They underwent no specific cardiac treatment.

Among the confirmed COVID-19 positive cases, two patients developed shortness of breath and received oxygen therapy, two were managed in ICU because of cardiac complications and were treated with inotropic drugs (one received ECMO implantation), and three had pneumonia for which one was treated with CPAP therapy (Figure 3). No death was reported in either group (Figure 3), and the rate of discharge from the hospital was 100%.

## 4. Discussion

Based on the sources of data used in this study, patients—both children and adults—with CHD were infected with COVID-19 and were admitted to Italian CHD Units between 21 February–4 April.

To the best of our knowledge, this is the first study on COVID-19 including patients with congenital heart disease.

We reported detailed cardiovascular information on seventy-six COVID-19-infected patients with CHD, with a particular focus on the number of cardiovascular complications, underlying cardiovascular comorbidities/risk factors, and fatal outcomes.

In the current study, among the 76 patients with COVID-19, 9% exhibited heart failure, which was the most common cardiovascular complication, followed by palpitations/arrhythmias (3%), stroke/TIA (3%), pulmonary hypertension (3%), and myocardial injury (1%).

Furthermore, when CHD-Covid-19+ patients were considered alone (Table 2), heart failure was observed in 55% of the total, while palpitations/arrhythmias, stroke/TIA and pulmonary hypertension were observed in 22% of cases.

In agreement with study from Guo et al. [5], demonstrating that patients with underlying CVD and other comorbid conditions are more prone to experience myocardial injury during the course of COVID-19, we also observed a high amount of myocardial involvement in CHD COVID-19-infected patients by summing-up those with myocardial injury and those with overt heart failure.

Moreover, similar to other previous reports from the general population [4,16,17], when analysing cardiovascular comorbidities and/or risk factors from our CHD patients, cardiovascular comorbidities other than CHD were relatively common in adults and were absent in the paediatric population. In fact, atrial fibrillation and obesity accounted for 9%, hypertension for 7%, and diabetes for 1% of the total.

The child group comprised two girls and two boys, with a mean age of 0.9 years. On the contrary, the adult group, with a mean age of 36.6 years, included 38 men and 34 women, in line with previous studies showing higher percentages of infection in men than in women [4,17].

Finally, against what has been reported to date on patients with cardiovascular disease [4,5,6], no deaths were observed in the whole cohort of patients with CHD; most of them presented with mild infection, without any severe symptoms or complication. The implications of the results in relation to general or immunological COVID-19 therapies [18] will be considered in an extended study.

Given the tremendous number of infections reported in Italy [19], the number of COVID-19-infected patients with CHD identified was relatively limited, especially if we consider the high CHD case load per year (about 300 per year for each) admitted to Italian CHD Units participating in our survey.

Some potential explanations might be speculated. Firstly, this phenomenon may be partially due to reduced chances of exposure of those patients, or incomplete recognition due to mild or asymptomatic disease, rather than resistance to infection. In our experience, families tend to be very protective of children or young adults with a history of CHD, and this might also play a role in reducing the exposure of CHD patients. Additionally, the Italian Government imposed a strict lockdown during the accelerating phase of the pandemic [20]. Consequently, patients with CHD together with those with comorbidities were advised to protect themselves from potential exposure to the severe acute respiratory syndrome coronavirus 2 (SARS-CoV-2) even more than the general population.

While it is true that congenital heart disease affects a relatively younger population, therefore less at risk for COVID-19 adverse outcomes, on the other hand, we did not observe, in our cohorts of congenital heart disease patients, the same critical respiratory outcomes and the higher mortality risk that have been previously described for cardiovascular diseases [4,5,6] other than CHD. These findings may suggest that that the occurrence of cardiovascular risk factors, like older age, obesity, hypertension and diabetes, which is much lower in CHD than that reported in studies on the general population [21], may play a major role in determining COVID-19 mortality, rather than cardiac disease per se. However, further evidence is awaited, as are randomised control trials with larger populations to confirm our observations.

### Study Limitations

The main limitation of the study was the possible lack of inclusion of asymptomatic patients. Even though our survey was conducted systematically through eight high-volume Italian CHD Units, covering different geographical areas, the epidemic has been spreading rapidly, and the incomplete identification of infected patients is possible.

## 5. Conclusions

Despite several reports suggesting that SARS-CoV-2 may have a more severe clinical presentation and higher fatality rate, in patients with cardiovascular co-morbidities, most of the patients included in our observational cross-sectional study presented an overall mild clinical course, and there were virtually no deaths for the congenital heart disease patients. Although the underlying reasons are not yet understood, these results are reassuring in the current literature panorama reporting an association between cardiovascular risk factors and the fatality rate in COVID-19 patients.

## Figures and Tables

**Table 1:** Clinical characteristics of patients with Congenital Heart Disease and COVID-19 infection.

	Total (*n* = 76)	suspCHD-Covid-19 (*n* = 67)
Age (years)	34.7	30
Sex (female, *n*)	36 (47%)	32 (48%)
Hypertension	5 (7%)	5 (7.5%)
Diabetes	1 (1%)	0
Obesity	7 (9%)	5 (7.5%)
Smokers	1 (1%)	1 (1.4%)
Pulmonary hypertension	2 (3%)	1 (1.4%)
AF/atrial tachicardia	7 (9%)	6 (9%)
Previous cardio-embolic stroke	2 (3%)	2 (3%)

Values are mean or *n* (%).

**Table 2:** Characteristics of 9 patients with Congenital Heart Disease infected with COVID-19.

	Patients								
Characteristics	1	2	3	4	5	6	7	8	9
Age (years)	35	30	30	0.3	48	52	47	2	1
Sex	Female	Male	Male	Male	Male	Female	Male	Female	Female
CHD	Univentricular heart s/p TCPC	TGA	VSD	Multiple	TGA (Mustard)	pAVSD	Ao stenosis; COA	PAx/VSD	TGA; PAx/VSD
		Atrial switch							
		LV-PA conduit		VSD (PAB)					
CV risk factors	None	None	Obesity	None	DM, Obesity	None	None	None	None
CV comorbidity	None	None	None	None	AF	PH, PLE, AV block	HF	None	None
CV complications	None	None	Chest pain	MyoInj; PEE; HF	HF	Stroke, Arrhythmias	Stroke, arrhythmias, worsening HF; ECMO implantation	HF; PH	HF; PH
Death (yes/no)	No	No	No	No	No	No	No	No	No

CHD = congenital heart disease; CV = cardiovascular; TCPC = total cavopulmonary connection; TGA = transposition of the great arteries; LV = left ventricle; PA = pulmonary artery; PAx = pulmunary atresia; VSD = ventricular septal defect; pAVSD = partial atrioventricular septal defect; PAB = pulmonary artery banding; DM = diabetes mellitus; AF = atrial fibrillation; PH = pulmonary hypertension; PLE = pleural effusion; HF = heart failure; MyoInj = myocardial injury; PEE = pericardial effusion; COA = coarctation of the aorta.
